# Evaluating the indicators of a heart rate variability analysis in dogs using Poincaré plots

**DOI:** 10.17221/49/2023-VETMED

**Published:** 2024-02-27

**Authors:** Taichi Kimura, Shunsuke Shimamura, Hiroshi Sakaya, Tetsuya Hayashi, Azusa Umemoto, Kosei Sakai, Masahiro Yamasaki, Tetsuya Hasegawa, Terumasa Shimada

**Affiliations:** ^1^Laboratory of Small Animal Clinical Medicine, Graduate School of Life and Environmental Sciences, Osaka Prefecture University, Izumisano, Osaka, Japan; ^2^R&D Division, Global New Business Development Unit, Smart Appliances & Solutions BU, SHARP CORPORATION, Yao, Osaka, Japan; ^3^Life Innovation & Material Laboratories, Corporate Research and Development Group, SHARP CORPORATION, Tenri, Nara, Japan; ^4^Laboratory of Small Animal Internal Medicine, Department of Veterinary Medicine, Faculty of Agriculture, Iwate University, Morioka, Iwate, Japan; ^5^Kakogawa Animal Hospital, Nishi-inokuchi, Higashi-kanki, Kakogawa, Hyogo, Japan

**Keywords:** canine, frequency-domain, geometric, Lorenz-plot, time-domain

## Abstract

Heart rate variability analyses using Poincaré plots can be useful for evaluating the autonomic nervous system function. However, the interpretation of the quantitative indicators of Poincaré plots remains controversial. Thus, few studies have verified the effectiveness of the quantitative indicators in veterinary medicine. This study aimed to verify the reliability of Poincaré plot indicators using pharmacological models in dogs. Four healthy beagles were used in this study. Each dog was treated with propranolol, atropine, and propranolol–atropine to block the sympathetic, parasympathetic, and sympathetic–parasympathetic functions, respectively. The quantitative indicators of the Poincaré plots were calculated based on data from 300 electrocardiogram beats collected before and after the administration of each drug and statistically analysed. The quantitative indicators of the Poincaré plots, such as the standard deviation perpendicular to the major axis (SD1), standard deviation along the major axis (SD2), and SD1 × SD2, significantly decreased after the drug administration in both the parasympathetic and sympathetic–parasympathetic blockade models. However, no significant differences were observed in SD1/SD2 between the groups. The Poincaré plots reflected the changes in the autonomic nervous system of dogs. In dogs, SD1, SD2, and SD1 × SD2 can detect a state in which parasympathetic nerve activity is suppressed.

Heart rate variability (HRV) is commonly used to analyse the activity and balance of the autonomic nervous system and can be examined using non-invasive heart rate (HR) monitors. The differences in the lengths of the beat-to-beat intervals are of special interest, as they depict HR fluctuations as markers for several influencing factors, such as physical activity, emotional stress, and neuroendocrinological processes. These interval variations are markers of the ability to regulate internal and external processes. HRV has been clinically applied in human medicine to assess cardiovascular diseases such as myocardial infarction (Casolo et al. 1992). It has also been reported as a useful stress marker (Kim et al. 2018).

The clinical application of HRV may be effective in evaluating various veterinary medical situations. However, this approach has not yet reached a practical application stage. One reason for this is the difficulty in obtaining acceptable data without artefacts because animals cannot control their behaviour during measurements, even if special devices such as Holter electrocardiograms (ECGs) are used (Blake et al. 2018). To make an HRV analysis practical for animals, it is necessary to develop highly accurate biometric sensors and artefact-tolerant analytical methods.

Poincaré plots are a geometric method used for HRV analysis. They can identify artefacts and outliers by visually evaluating plot patterns (Guzik et al. 2007). Therefore, Poincaré plots may be appropriate for the HRV analysis in studies involving sample sizes of animals. In the Poincaré plot analysis, the temporal distance between two adjacent electrocardiogram (ECG) R-waves is taken as the RR-interval (RRI), and two consecutive RRIs are defined as RRI_n_ and RRI_n+1_ and plotted continuously on the XY plane as (RRI_n_, RRI_n+1_). This analysis can visually evaluate changes in the autonomic nervous activity based on plot patterns and quantify them by determining the degree of dispersion of the plots (Guzik et al. 2007; Blake et al. 2018).

To evaluate HRV using Poincaré plots, the following four indicators are commonly used in human medicine: SD1 [the standard deviation measuring the dispersion of points in the plot perpendicular to the major axis (Kamen et al. 1996; Brennan et al. 2002)]; SD2 [the standard deviation measuring the dispersion of points along the major axis (Brennan et al. 2002; Guzik et al. 2007)]; SD1/SD2 (Toichi et al. 1997; Hoshi et al. 2013); and SD1 × SD2 (Toichi et al. 1997; Guzik et al. 2007). However, these indicators were designed to quantify human plot patterns. It has been reported that dogs could show different plot patterns from humans (Blake et al. 2018; Moise et al. 2020). Therefore, it remains unclear whether these quantitative indicators could be extrapolated directly to dogs. The interpretation of these indicators in dogs should be verified independently. To the best of our knowledge, there have been no detailed evaluations of these indicators in veterinary medicine.

This study aimed to validate the quantitative indicators of Poincaré plots in dogs, for which differences in plot patterns among species have been reported. We suggest that clarifying the validity of the quantitative indicators in dogs would lead to the widespread use of HRV analyses in veterinary medicine.

## MATERIAL AND METHODS

### Animals

The study included four healthy beagles (two females and two males) aged 5–10 years (mean 6.25 ± 2.2 years), and weighing between 11.3 and 16.5 kg (mean 13.6 ± 1.9 kg). All the dogs underwent physical examination and general blood testing and were confirmed to be healthy. The Animal Care and Use Committee of our university approved the study protocol (Approval No. A201649).

### Equipment

To obtain the ECG data used for HRV analysis, we developed an ECG sensor device with fewer restrictions to reduce the effects of the physical and mental stress on the results. This device was a small rucksack type and an ECG sensor (6.3 × 5.8 × 1.6 cm in size and 50 g in weight, including the body surface temperature sensor, battery, and battery case) mounted in the back pouch.

The ECG data were obtained using electrodes placed on the belts of both axillary portions and collected at a sampling rate of 100 Hz using filters to remove any electrical noise. The sensor had a built-in transmitter. The data was transmitted wirelessly and stored in a computer for processing. The R-peaks of the ECG signals were detected based on the signal amplitude and peak spacing. The threshold amplitude and interval were predetermined. The RRI was obtained as the interval between two consecutive R peaks. Artefacts in the collected ECG information can cause significant distortions in the HRV analysis results. Typical artefacts include missing, redundant, or inconsistent beat detection, and ectopic beats such as ventricular extrasystoles and other arrhythmias.

Here, we scrutinised all the measured RRI values to correct for the artefacts and ectopic beats. Artefacts were detected according to the algorithm of general analysis software (Amaya et al. 2020). Those identified as having more than twice the length of the RRI of the previous beat or shortened by more than half were excluded from the analysis. The dogs were simultaneously equipped with a device and Holter electrocardiograph (HS1000 standard; Fukuda ME Kogyo, Tokyo, Japan). An accuracy verification experiment was conducted in advance. Regarding the HR measurements, the two systems showed an 87% correlation (*R* = 0.868 847) after 180 min of recording.

### Experimental protocol

The experimental protocol was based on previous reports (Toichi et al. 1997; Palazzolo et al. 1998). The dog cage (60 × 72 × 55 cm) used in this experiment was placed in a quiet room. A wearable device was mounted on each dog, and an intravenous catheter was placed in the right cephalic vein for drug administration. All the experiments were performed individually. A 30-min acclimation period was provided, followed by a 20-min period for the ECG measurement as a baseline. Subsequently, the drugs that act on the autonomic nervous system were administered according to the following protocols: First, propranolol hydrochloride (Nichi-Iko Pharmaceutical, Toyama, Japan) was administered [0.1 mg/kg, intravenously (i.v.)] as a sympathetic nerve blockade model. Second, atropine sulphate [Fuso Pharmaceutical Industries, Osaka, Japan (0.05 mg/kg, i.v.)] was administered as a parasympathetic nerve blockade model. Third, atropine sulphate (0.05 mg/kg, i.v.), was administered as a sympathetic-parasympathetic nerve blockade model 5 min after the administration of propranolol hydrochloride.

The ECG data were obtained 30 min after the atropine administration. One of the three aforementioned administration protocols was performed once daily for each dog. All the protocols were applied to all the dogs.

### Data analyses

After filtering out the noise, the RRI data of 300 beats immediately before the administration and 300 beats counted 5 min after the administration were analysed. The analysis was performed using commercial software (R Foundation for Statistical Computing, Vienna, Austria). Furthermore, the quantitative indicators using the HRV analysis were calculated. In terms of the Poincaré plots, the following four indicators were used: SD1, SD2, SD1/SD2, and SD1 × SD2 (Kamen et al. 1996; Toichi et al. 1997; Brennan et al. 2002; Guzik et al. 2007; Hoshi et al. 2013). In the time-domain analysis, three indicators were used: the SD of all the RRIs (SDNN), the percentage of normal RR intervals that differed by 50 ms (PNN50), and the root mean square of the successive RRI differences (RMSSD) (Guzik et al. 2007). In the frequency-domain analysis, the following three indicators were used: low frequency (LF; the integrated value of the spectral intensity between 0.04 and 0.15 Hz), high frequency (HF; the integrated value of the spectral intensity between 0.15 and 0.4 Hz), and LF/HF (Guzik et al. 2007). These values were compared before and after the drug administration.

### Statistical analyses

Statistical analyses were performed using commercially available software (R Foundation for Statistical Computing, Vienna, Austria). All the data were analysed using non-parametric methods due to the small sample size. Therefore, all the statistical comparisons before and after the drug administration in each group were performed using the Wilcoxon signed-rank test. Descriptive statistics are presented as medians and ranges for all the data. Statistical significance was accepted when the test statistic was zero (*T* = 0).

## RESULTS

**Figure 1:**
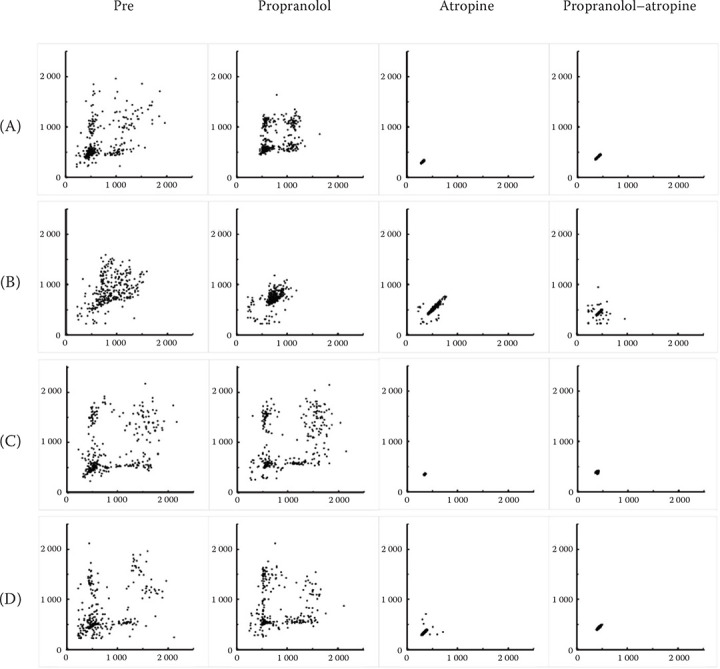
Poincaré plots in the individuals before and after each drug administration (A–D) Dogs 1, 2, 3, and 4, respectively. The plots for each dog are arranged from left to right in the following order: Before administration, after the propranolol administration, after the atropine administration, and after the propranolol–atropine administration. After the propranolol administration, the plot is more dispersed in all directions, with a clear “cloud” pattern seen in the upper right. Individual differences in the degrees of dispersion were observed. After the administration of atropine and propranolol–atropine, the plot converges at approximately 500 ms

In all the experiments, sufficient ECG data were obtained for the analysis. A scatter plot was created using Poincaré plots based on the analysed data (Figure 1).

Although there were some differences in the plot variability among the dogs, the overall trend was consistent. After the propranolol administration, the plot tended to disperse in all directions compared with that before the drug administration, and the variation was small. After the atropine administration, the plot tended to converge to the y = x-axis and in the range of 200–1 000 ms compared with that before the administration. This tendency was also observed when atropine was administered following propranolol.

Table 1 presents a comparison of the HR, mean RRI, and RRI variance regarding each drug before and after administration.

**Table 1:** Variables derived from the electrocardiograms in the dogs before and after the drug administration

Variables in each protocol	Pre			Post		*T*
	median	range		median	range	
Propranolol						
HR (beats/min)	82.61	66.91~94.74		75.48	61.22~84.11	0.00^a^
Mean RRI (ms)	726.13	631.37~899.63		795.47	713.37~979.33	0.00^a^
RRI variance (× 10^3^)	117.63	26.72~238.83		110.49	22.76~231.19	3.00
						
Atropine						
HR (beats/min)	81.85	55.90~87.80		171.44	113.21~189.47	0.00^a^
Mean RRI (ms)	732.80	685.30~751.77		351.27	318.00~530.13	0.00^a^
RRI variance (× 10^3^)	104.32	36.98~200.87		0.80	0.06~6.72	0.00^a^
						
Propranolol–atropine						
HR (beats/min)	76.41	67.67~109.09		134.85	132.35~142.86	0.00^a^
Mean RRI (ms)	682.45	548.50~885.30		430.75	398.02~455.10	0.00^a^
RRI variance (× 10^3^)	38.86	0.44~78.78		0.38	0.01~3.75	0.00^a^

All the data are expressed as the median and range

^a^Value is significantly (*T* = 0) different before and after the drug administration

HR = heart rate; RRI = RR interval

After the propranolol administration, the HR significantly increased and the mean RRI significantly decreased; however, there was no significant difference in the RRI variance compared to the values before the administration. After the atropine and propranolol–atropine administration, the HR significantly increased and the mean RRI and RRI variance significantly decreased.

The results of the geometric analysis indicators (e.g., SD1, SD2, SD1/SD2, and SD1 × SD2) before and after the drug administration are shown in Table 2.

**Table 2:** Comparisons of the geometric analysis indicators before and after the drug administration

Indicators in each protocol	Pre			Post		*T*
	median	range		median	range	
Propranolol						
SD1 (ms)	388.81	198.03~557.70		347.98	187.83~558.58	4.00
SD2 (ms)	250.25	119.86~403.80		302.22	101.67~388.86	3.00
SD1/SD2	1.56	1.38~1.65		1.36	1.07~1.85	3.00
SD1 × SD2	311.92	154.06~474.55		323.96	138.19~466.06	4.00
						
Atropine						
SD1 (ms)	380.03	212.67~527.13		32.69	9.70~109.02	0.00^a^
SD2 (ms)	244.49	169.98~350.60		17.31	4.97~39.48	0.00^a^
SD1/SD2	1.47	1.25~1.66		2.28	1.45~4.63	1.00
SD1 × SD2	304.62	190.13~429.90		22.94	7.24~65.60	0.00^a^
						
Propranolol–atropine						
SD1 (ms)	305.30	185.55~532.83		35.21	14.76~64.60	0.00^a^
SD2 (ms)	194.40	120.86~330.15		7.83	5.44~57.77	0.00^a^
SD1/SD2	1.57	1.54~1.61		3.16	1.12~6.99	2.00
SD1 × SD2	243.62	149.75~419.42		14.73	11.29~61.09	0.00^a^

All the data are expressed as the median and range

^a^Value is significantly (*T* = 0) different before and after the drug administration.

SD1 = deviation perpendicular to the major axis; SD2 = standard deviation along the major axis

No significant differences were observed in any of the indicators before or after the propranolol administration. All the indicators, excluding SD1/SD2, showed a significant decrease after the atropine and propranolol–atropine administration compared with their levels before the administration. The results of the time-domain analysis indicators (e.g., SDNN, pNN50, and RMSSD) before and after each drug administration are shown in Table 3. No significant differences were observed in any of the indicators before or after the propranolol administration. All the indicators showed a significant decrease after the atropine and propranolol–atropine administration compared with those before the administration. The results of the frequency-domain analysis indicators (e.g., LF, HF, and LF/HF) are presented in Table 4.

**Table 3:** Comparisons of the time-domain analysis indicators before and after the drug administration

Indicators in each protocol	Pre			Post		*T*
	median	range		median	range	
Propranolol						
SDNN (ms)	326.64	163.46~488.70		326.20	150.85~480.82	3.00
PNN50 (%)	74.08	65.89~81.27		76.59	62.88~84.62	2.00
RMSSD (ms)	353.91	169.50~571.09		427.40	143.78~549.93	3.00
						
Atropine						
SDNN (ms)	320.25	192.30~448.19		26.49	7.85~82.03	0.00^a^
PNN50 (%)	78.09	70.90~90.97		1.17	0.00~7.02	0.00^a^
RMSSD (ms)	345.78	240.39~495.82		24.48	7.04~55.83	0.00^a^
						
Propranolol–atropine						
SDNN (ms)	255.61	156.74~444.10		25.42	12.08~61.21	0.00^a^
PNN50 (%)	73.24	58.53~84.62		0.00	0.00~10.37	0.00^a^
RMSSD (ms)	274.93	170.93~466.92		11.08	7.69~81.70	0.00^a^

All the data are expressed as the median and range

^a^Value is significantly (*T* = 0) different before and after the drug administration

PNN50 = percentage of normal RR intervals that differed by 50 ms; RMSSD = root mean square of the successive RR interval differences; SDNN = standard deviation of all the RRIs

**Table 4:** Comparisons of the frequency-domain analysis indicators before and after the drug administration

Indicators in each protocol	Pre			Post		*T*
	median	range		median	range	
Propranolol						
LF (× 10^8^ ms^2^)	397.00	130.00~1 230.00		436.00	121.00~1 540.00	4.00
HF (× 10^8^ ms^2^)	2 950.00	136.00~7 490.00		2 500.00	105.00~8 120.00	5.00
LF/HF	0.24	0.08~1.24		0.22	0.15~1.15	4.00
						
Atropine						
LF (× 10^8^ ms^2^)	475.00	189.00~626.00		0.99	0.01~24.30	0.00^a^
HF (× 10^8^ ms^2^)	1 490.00	434.00~4 330.00		1.55	0.02~6.02	0.00^a^
LF/HF	0.35	0.12~0.44		2.30	0.15~19.01	1.00
						
Propranolo–atropine						
LF (× 10^8^ ms^2^)	246.00	56.90~1 390.00		0.61	0.13~7.00	0.00^a^
HF (× 10^8^ ms^2^)	719.00	146.00~7 530.00		0.88	0.08~6.00	0.00^a^
LF/HF	0.35	0.18~0.39		1.35	0.42~3.02	0.00^a^

All the data are expressed as the median and range

^a^Value is significantly (*T* = 0) different between the pre- and post-administration of the drug

HF = high frequency; LF = low frequency

No significant differences were observed in any of the indicators in the sympathetic blockade model. The LF and HF were significantly decreased after the atropine and propranolol–atropine administration compared to their levels before the administration. The LF/HF significantly decreased only when atropine was administered after the propranolol administration compared with that before the administration.

## DISCUSSION

In recent years, research on HRV analyses has also been conducted in the veterinary field, in line with the development of HRV research in the medical field (Calvert and Jacobs 2000; Oliveira et al. 2012; Rasmussen et al. 2012; Zupan et al. 2016; Bogucki and Noszczyk-Nowak 2017; Broux et al. 2017). A report on Poincaré plots suggested that it was suitable for the analysis of animals. Notably, different plot patterns have been observed between humans and dogs (Blake et al. 2018; Moise et al. 2020). Therefore, this study aimed to create a pharmacological model and verify the practicality of the autonomic nerve activity index in the Poincaré plots of dogs.

Sympathetic, parasympathetic, and sympathetic–parasympathetic blockade models were created as previously described (Toichi et al. 1997; Palazzolo et al. 1998; Du et al. 2017). All the pharmacological autonomic nervous blockade models were adequately prepared based on changes in the HR and mean RRI. The changes in these parameters in the sympathetic blockade model were significant, but small. This might be related to the basal autonomic nerve activity in dogs. Under normal conditions, the parasympathetic nerve predominates in dogs compared with humans (Moise et al. 2020). Thus, the sympathetic blockade is less likely to be vital in healthy dogs.

The HR of the sympathetic–parasympathetic blockade model in the present study (136 ± 4 beats per min) was close to, but slightly higher, than that of the intrinsic sinus rhythm in adult dogs according to a previous study using the same drugs (120 ± 9 beats per min) (Du et al. 2017). Since the previous study included dogs under general anaesthesia, our results of dogs that were awake may have shown a slightly higher HR.

The indicators of the time- and frequency-domain analyses, used in previous studies, were calculated to compare the autonomic nerve activity in the present study. Most indicators showed significant changes during the parasympathetic and sympathetic–parasympathetic blockade. The LF/HF ratio did not show any statistical significance during the parasympathetic blockade. However, the median value was higher during the parasympathetic blockade than during the sympathetic–parasympathetic blockade, suggesting the possibility of a type II error. Therefore, we confirmed that the changes in the autonomic nerve activity in the pharmacologically produced model were reflected in the time- and frequency-domain analyses.

One of the problems encountered when interpreting the results of HRV analyses is the activation of the sympathetic nerve activity by disturbing factors such as exercise or emotional changes. The advantage of Poincaré plots over other methods, such as time- and frequency-domain analyses, is that outliers and artefacts could be easily identified (Guzik et al. 2007). Plots that deviate from the overall trend could be visually recognised as artefacts or excluded using analytical algorithms. In the present study, the basal plot patterns tended to be similar to those in previous reports including those in healthy dogs (Blake et al. 2018; Moise et al. 2020). Moreover, the convergence pattern of the plot after the drug administration was consistently observed in all the dogs with a parasympathetic blockade. It may be possible to confirm the validity of the data by verifying the plots that deviate from these patterns.

SD1, SD2, SD1/SD2, and SD1 × SD2 are quantitative indicators of the HRV analysis in Poincaré plots. Guzik et al. (2007) reported that SD1 and SD2 could reflect the parasympathetic activity and both sympathetic and parasympathetic activities, respectively. They also revealed that SD1/SD2 was used as an index of the balance between the sympathetic and parasympathetic nerve activities, and SD1 × SD2 was used as an index of the total HRV (Guzik et al. 2007). However, these indicators were based on the typical linear pattern of Poincaré plots in humans (Brennan et al. 2001; Moise et al. 2020). There is no evidence that these indicators could be applied directly to dogs with plot patterns that differ from those in humans (Blake et al. 2018; Moise et al. 2020). Furthermore, Toichi et al. (1997) reported that *T* (4 × SD1), which is mathematically equivalent to SD1, is affected by both sympathetic and parasympathetic nerve activities. In addition, Rahman et al. reported that Poincaré plots could reflect changes in the parasympathetic activity rather than in the sympathetic activity (Toichi et al. 1997; Rahman et al. 2018). Overall, the interpretation of quantitative indicators of Poincaré plots is inconsistent, even in human medicine. Hence, this should be verified in dogs when an HRV analysis is performed using Poincaré plots.

In this study, SD1, SD2, and SD1 × SD2 significantly decreased after the parasympathetic and sympathetic–parasympathetic blockade. However, no significant changes in SD1/SD2 were observed before or after the drug administration. In resting dogs, the dispersion of the plot along the y = –x axis was large, indicating a square-shaped plot pattern. Therefore, SD1/SD2 is expected to be close to 1. Similarly, after the parasympathetic blockade, the plots converged, and the ratio was also expected to be close to 1. Notably, SD1/SD2 was close to 1 for each model in this study. Therefore, SD1/SD2 may not be interpreted in dogs in the same manner as in humans. The results showed that SD1, SD2, and SD1 × SD2 could be used to evaluate the parasympathetic nerve activity.

Although the HR and mean RRI changed significantly in the sympathetic blockade model, no indicators could be detected in the present study. Existing indicators of HRV, such as the sympathetic blockade model, cannot evaluate subtle changes. This indicates that slight variations in the autonomic nerve activity in daily life may not be detected using these indicators. However, subtle differences in the plot dispersion before and after the propranolol administration were observed. For instance, a “cloud” pattern and “zone of avoidance”, which indicates parasympathetic dominance, became more clearly visible in the sympathetic blockade model (Moise et al. 2010; Moise et al. 2020). Thus, it is possible that the autonomic nerve activity can be evaluated in more detail by combining quantification indicators and geometric pattern analysis; however, further research is needed.

In this study, 300 data beats were used for each analysis. To obtain a consistent number of Poincaré plots for analysis, the number of beats rather than the time unit was selected. The following were the reasons for setting the number of samples to 300 beats: first, there were pharmacological limitations.

It has been reported that the serum concentration of atropine decreases rapidly within 10 min of intravenous administration (Berghem et al. 1980). Longer measurement times can compromise the reliability of the measured data. Thus, we considered that the analysis of 300 beats, which can finish measurements within 10 min including the acclimation time, is adequate. Second, it is difficult to obtain long-term resting data in clinical practice. The results of the analysis at 300 beats in this study were considered to completely reflect the autonomic nervous activity of the model, indicating its potential use in shorter measurements in clinical practice. If the number of Poincaré plots is extremely small, visual patterns may not be accurately observed. However, all the data samples in this study were able to identify specific plot patterns, indicating that the 300 beat measurement could be analysed without problems.

There are several disturbance factors in HRV. It is reported that the breed, weight, and age can affect the results of HRV in dogs (Doxey and Boswood 2004; Blake et al. 2018; Moise et al. 2020). In addition, the influence of sex on the autonomic nerve activity has been reported in humans (Voss et al. 2015). Although these factors were not verified in this experimental study, further clinical research is needed to determine their effect on the indicators of Poincaré plots.

This study was conducted using a pharmacologically prepared canine model. Although significant changes were observed, caution should be exercised when applying these results in clinical cases. Changes in the autonomic nervous system activity in daily life are expected to be more subtle than the pharmacological changes observed in this study. For the practical application of an HRV analysis in dogs, it is necessary to verify the changes under more clinically stressful conditions, even if each indicator is already in practical use in humans. In addition, the number of dogs included in this study was small. Results that were not statistically significant may become more significant with a larger sample size. However, the plot patterns at baseline in the present study were similar to those previously reported (Blake et al. 2018; Moise et al. 2020). Furthermore, the R2 values, indicating the model fit, for all the indicators of Poincaré plots and the time-domain analysis at the parasympathetic and sympathetic–parasympathetic blockade were acceptable (> 0.7), but were low for the sympathetic blockade. This finding suggests that a larger sample size is unlikely to alter the major results.

Poincaré plots for the HRV analysis were evaluated using canine sympathetic, parasympathetic, and sympathetic–parasympathetic blockade models. The quantitative indicators of the Poincaré plots, except for SD1/SD2, reflect the parasympathetic and sympathetic-parasympathetic blockade states. Furthermore, the Poincaré plots provided visual information for each blocking state. In conclusion, the Poincaré plot is expected to become an analytical option for HRV analysis, similar to time- and frequency-domain analyses.
